# How Is Endodontics Taught in Italy? A Survey of Italian Dental Schools

**DOI:** 10.3390/jcm11237190

**Published:** 2022-12-02

**Authors:** Giovanni Mergoni, Irene Citterio, Andrea Toffoli, Guido Maria Macaluso, Maddalena Manfredi

**Affiliations:** Centro Universitario di Odontoiatria, Dipartimento di Medicina e Chirurgia, Università di Parma, 43126 Parma, Italy

**Keywords:** endodontics, undergraduate training, dental education, online survey, Italy

## Abstract

The aim of our study was to investigate how endodontics is taught in Italian universities. An online survey was conducted from August to December 2021. A comparison between courses led by full or associate professors (Group 1) versus courses led by other figures, such as researchers or temporary lecturers (Group 2), was made. A total of 28 out of 36 schools participated (78%). In most schools, endodontics is taught in the fifth year to 15–29 students. All schools planned pre-clinical endodontic training, and in 25/28 schools (89.3%), clinical endodontic training was also provided. The course programs varied among schools, and significantly more hours were allocated to teaching nonsurgical root canal treatment in Group 1 schools than in Group 2 schools. The average numbers of hours of preclinical and clinical training were 34.3 ± 23.6 and 84.1 ± 76.7, respectively. All schools used rotary NiTi files in their clinical training, and the vertical condensation of hot gutta-percha was the most frequently taught obturation technique. As expected, the scenario of endodontic education in Italian universities was variable and needs harmonization. Courses led by full or associate professors seem to be better structured.

## 1. Introduction

Root canal treatment (RCT) aims to cure or prevent apical periodontitis. Pulp and periapical diseases have a high worldwide prevalence among the adult population [1], and, since the speciality of endodontics is still not established in Italy [2], RCT is one of the most frequent interventions delivered by general dentists [3].

Consistent with what has been observed in other European countries [4,5,6,7], the quality of endodontic treatments performed by Italian general dentists is, in many cases, suboptimal [8,9,10]; a possible reason could be the insufficient acquisition of adequate knowledge and technical skill during undergraduate training.

In 2013, in order to promote standards of scientific education and clinical training, the European Society of Endodontology (ESE) updated their undergraduate curriculum guidelines [11]. The ESE guidelines aim to guide dental schools in designing their endodontic curricula to achieve learning objectives in three different domains: scientific foundations of endodontic practice, nonsurgical endodontic treatment, and surgical endodontic treatment. At the end of the 6-year training period, the graduating dentist is expected to be able to deal with nonsurgical endodontic treatments of uncomplicated teeth.

In Italy, the system of dental education confers autonomy to each of the 37 schools in defining the course programs aimed at achieving the agreed level of competency. That implies some variability in endodontic study plans between universities. In addition, the academic qualifications of the person in charge of the course can vary among the schools. He or she could be a permanent-structured academic figure, such as a full professor or an associate professor, or a nonpermanent-structured figure, such as a researcher or a temporary lecturer.

The aim of our study was to investigate how endodontics is taught in Italian universities and to determine whether the academic qualifications of the person in charge of teaching affect the training program.

## 2. Materials and Methods

We conducted a survey inviting the undergraduate endodontic program leaders of all the Italian dental schools, with the exclusion of the recently established UniCamillus University in Rome, as the endodontic course had not yet been provided at the time of the survey. We used the questionnaire, first proposed by Al Raisi et al. in the UK [12] and then by Segura-Egea et al. in Spain [13], after being modified and translated. After consulting members of the staff involved in endodontic education in our institution, questions #13, #18, #21, and #23 of the original questionnaire were eliminated. Question #13 queried the degree of the case complexity of root canal treatments performed in clinical training according to the American Association of Endodontists (AAE) assessment tool [14] and was not included since this tool is not widely used in Italy. Question #18 was an open-ended query about the method of root canal preparation used. This question would require an elaborate answer that hardly suits an online questionnaire. In addition, information about the method of root canal preparation could be obtained from other questions included in the questionnaire. Question #21 asked for advanced endodontic materials used in preclinical and clinical endodontic training. This question was not included as the definition of “advanced material” is rather vague and can be misunderstood by the respondent. Question #23 queried the type of restoration undertaken after the completion of a root canal treatment. This question was not included because it was beyond the scope of the current survey, and, in addition, the type of restoration is usually chosen on a case-by-case basis according to the clinical findings. Question #6 (What are the qualifications of the person in charge of the course?), Question #9 (What is the average number of students attending the course?), and Question #15 (What is the average cost in euros that each student has to pay to purchase the material to be used in the preclinical training?) were added because they were considered important for outlining the undergraduate endodontic program. The final version of the questionnaire included 32 multiple-choice or open-ended questions on the following subjects: organization of the course, timing, preclinical and clinical training, teaching methods, topics covered, teaching staff, armamentarium, and techniques used (see the Supplementary Materials).

The email addresses of the endodontic course leaders were found online on the universities’ websites or via personal contacts.

The people in charge of the endodontic courses were emailed in September 2021 to explain the objectives of the survey and to invite them to anonymously participate using Google Forms (Google LLC, Montain View, CA, USA). An invitation reminder was emailed after 3 weeks. One of the authors, G.M., who leads the endodontic course at Parma University, filled out the questionnaire as well.

### Statistical Analysis

The results were obtained directly from Google Forms, maintaining anonymity, and analysed using Microsoft Excel 15.13.3 (Microsoft Corporation, Redmond, WA, USA) and Prism 4.01 (GraphPad software, San Diego, CA, USA). Based on the answer to Question #6 about the qualifications of the course leader, the schools were divided into two groups: Group 1, in which the leader was a full professor or an associate professor; and Group 2, in which the course leader was another figure. Continuous data were reported as the mean and standard deviation (SD), and they were compared with the Mann–Whitney U test. Categorical data were reported as a frequency and a percentage, and they were compared using Fisher’s exact test. *p* < 0.05 defined statistical significance. Taking into account the descriptive purpose of the study, it was judged as unnecessary to adjust for multiple comparisons [15].

In three schools, clinical training was not provided; thus, for questions regarding clinical training, frequencies and means were calculated on a total of 25 schools. One school, belonging to Group 2, that provided clinical training did not answer question #32 (regarding the minimum number of root canals to be treated by students during clinical training), so the proportions were calculated for a total of 24 schools.

## 3. Results

A total of 28 out of 36 schools responded to the survey (78%). Group 1 was composed of 12 schools; in 6 of them, the course leader was a full professor, and in the remaining 6, it was an associate professor. Group 2 was composed of 16 schools; in 4 schools, the course was led by a researcher, and in 12, it was led by a temporary lecturer.

### 3.1. General Information

**Figure 1:**
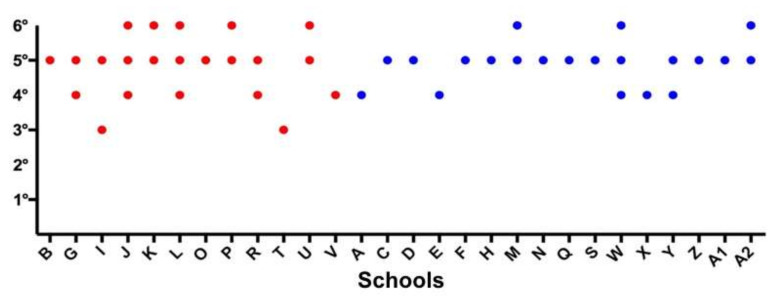
Answers to the question “In which year/years is endodontics taught?” Schools belonging to Groups 1 and 2 are represented by red and blue dots, respectively.

Endodontics was taught in 1 or more academic years. In most universities, the teaching was provided in the 5th year (23/28; 82.1%) and in the 4th year (10/28; 35.7%), while in some schools, the teaching occurred in the 6th year (8/28, 28.6%) and in the 3rd year (2/28, 7.1%). In no school was endodontics taught in the 1st or 2nd year. Endodontics was taught over several academic years in 8/12 (66.7%) and 4/16 (25%) schools in Group 1 and Group 2, respectively (*p* = 0.047) (Figure 1).

All schools (28/28, 100%) delivered lectures and preclinical training, and 25/28 schools (89.3%) also provided clinical endodontic training. The three universities with no clinical training belonged to Group 2.

**Figure 2:**
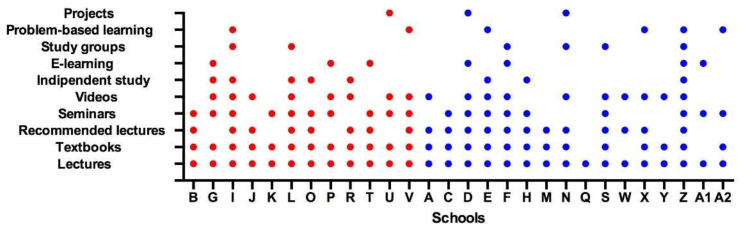
Answers to the question, “What teaching methods are used”? Schools belonging to Groups 1 and 2 are represented by red and blue dots, respectively.

While most of the schools used textbooks (25/28; 89.3%), recommended readings (20/28; 71.4%), seminars (19/28; 67.9%) and videos (18/28; 64.3%), a minority of schools reported independent study (9/28; 32.1%), e-learning (7/28; 25%), study groups (6/28; 21.4%), problem-based learning (6/28; 21.4%) and projects (3/28; 10.7%) (Figure 2).

The prevailing trend among schools was to teach topics related to the biological bases of endodontics (pulp histology, endodontic microbiology, root canal anatomy and pulp pathology, and endodontic radiology) in the 5th year or earlier, and to then treat topics related to the clinical treatment of endodontic diseases in the 5th or 6th year (see the Supplementary Materials).

**Figure 3:**
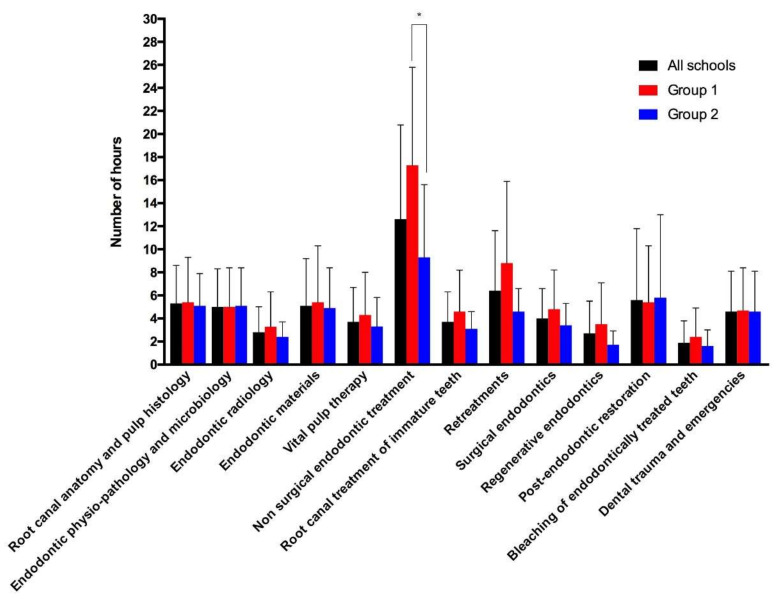
Hours allocated to the different endodontic topics. Black, red, and blue bars represent the mean number of hours ± 1 SD for all schools, Group 1, and Group 2, respectively (* *p* < 0.05).

The hours dedicated to teaching the different endodontic subjects are shown in Figure 3. The greatest number of hours were allocated to the topic “Nonsurgical root canal treatment” (12.6 ± 8.2), while the fewest number were allocated to the topic “Bleaching of endodontically treated teeth” (2.4 ± 2.5). Significantly more hours were allocated to teaching the topic “Nonsurgical root canal treatment” in Group 1 than in Group 2 (17.3 ± 8.5 in Group 1, and 9.3 ± 6.3 in Group 2, *p* = 0.0087). No significant differences were found when comparing the number of hours allocated to the other endodontic subjects in Groups 1 and 2.

In 14/28 schools (50%), the clinical activity of the course leader was limited to endodontics or endodontics and operative dentistry (6/12 in Group 1, and 8/16 in Group 2, not significant); in the remaining 14/28 schools (50%), the clinical activity of the course leader was general dentistry with a particular interest in endodontics (6/12 in Group 1, and 8/16 in Group 2, not significant).

The number of collaborators involved in the delivery of the course varied from 0 to 10, with an average of 2.2 ± 2.6 collaborators (3.5 ± 3.2 in Group 1, and 1.1 ± 1.4 in Group 2, *p* = 0.006). The profiles of the collaborators included tutors, temporary lecturers, postdoctoral fellows, researchers, and associate professors.

The number of students attending the courses in the different schools is given in Table 1. In a total of 7 out of the 12 schools in Group 1, and in 3 out of the 16 schools in Group 2, the number of students was ≥30 (*p* = 0.049).

### 3.2. Preclinical Training

The staff:student ratio during preclinical training ranged from 1:4 to 1:20, with an average ratio of 1:8.9 (1:7.6 in Group 1, and 1:10 in Group 2, not significant).

The number of hours dedicated to preclinical activities varied between schools, ranging from 4 to 100, with a mean of 34.3 ± 23.6 h (46.1 ± 28.2 in Group 1, and 24.1 ± 14.5 in Group 2, *p* = 0.059).

Students performed root canal treatments of single-rooted teeth, root canal treatments of multirooted teeth, retreatments, endodontic surgery, bleaching of endodontically treated teeth, and vital pulp therapy in 100%, 96.1%, 46.1%, 19.2%, 11.5%, and 7.7% of the schools, respectively. In no university did preclinical training include endodontic regeneration.

During the preclinical training, students used natural teeth, commercial plastic teeth, canals in acrylic resin blocks with simple curvatures, canals in teeth made by 3D printing, and canals in acrylic resin blocks with S-shaped curvatures in 82.1%, 46.4%, 39.3 %, 17.9%, and 7.1% of the schools, respectively.

The material to be used in the preclinical training was freely delivered in 41.7% of the schools. In the other schools, the cost ranged from EUR 30 to EUR 2000, with a mean of EUR 352 ± 524 (EUR 500 ± 709 in Group 1, and EUR 201 ± 268 in Group 2, not significant).

Manual plus rotary/reciprocating instruments were used in 22/28 (78.6%) schools; only manual instruments were used in 3/28 (10.7%) schools; and only rotary/reciprocating instruments were used in the remaining 3/28 (10.7%) schools.

No magnifying systems were used in 10/28 (35.7%) schools during preclinical training, and among the different magnifying systems, a microscope was used in 6/28 schools. Ultrasounds were used in 10/28 (35.7%) schools.

Only 4 schools taught a single obturation technique during preclinical training; the remaining 24 taught a combination of 2–7 techniques. The most-taught technique was vertical hot condensation, followed by the carrier-based technique, and the continuous wave of condensation technique.

A total of 17 out of the 28 schools (60.7%) (6/12 in Group 1, and 11/16 in Group 2, not significant) did not require a minimum number of root canals to be treated by students during preclinical exercises. In the remaining schools, the minimum number of root canals to be treated ranged from 1 to 15, with a mean of 7.5 ± 3.3 (8.2 ± 3.9 in Group 1, and 7.7 ± 3.5 in Group 2, not significant).

### 3.3. Clinical Training

The staff:student ratio during clinical training ranged from 1:2 to 1:20, with an average of 1:6.8 (1:6 in Group 1, and 1:7.5 in Group 2, not significant). The number of hours dedicated to clinical activity was highly variable and ranged from 0 to 250, with a mean of 84.1 ± 76.7 h (90.83 ± 74.35 in Group 1, and 78.43 ± 81.02 in Group 2, not significant). Students performed root canal treatments of single-rooted teeth, root canal treatments of multirooted teeth, retreatments, vital pulp therapy, bleaching of endodontically treated teeth, endodontic surgery, and endodontic regeneration in 91.7%, 79.2%, 50.0%, 45.8%, 29.2%, 12.5%, and 4.2% of the schools, respectively. In two schools (8.3%), students only assisted, without directly delivering the endodontic treatments.

In 17/28 schools (60.7%), there was a clinical area specifically assigned to endodontics (9/12 schools in Group 1, and 8/16 schools in Group 2, not significant).

A combination of manual and rotary/reciprocating instruments was used in 21/25 (78.6%) schools, and rotary/reciprocating instruments were exclusively used in 4/25 (21.4%). No school used exclusively manual instruments during clinical training.

No magnifying systems were used in 2/25 (8%) schools during clinical training, and a microscope was used in 8/25 schools. Ultrasounds were used in 21/25 (84%) schools.

In 23/25 (92%) schools, the working length was determined using both an electronic apex locator and X-rays, and in 2/25 (8%) an apex locator was used exclusively.

In 21/25 schools, sodium hypochlorite was used alone or in combination with EDTA, saline, tap water, local anaesthetic solution, chlorhexidine, hydrogen peroxide, or alcohol. In 19 schools, EDTA was used, always in combination with other irrigants. In four schools, sodium hypochlorite was not used: one school used only tap water, one school used tap water plus alcohol, one school used tap water plus saline, and one school used EDTA and saline.

**Figure 4:**
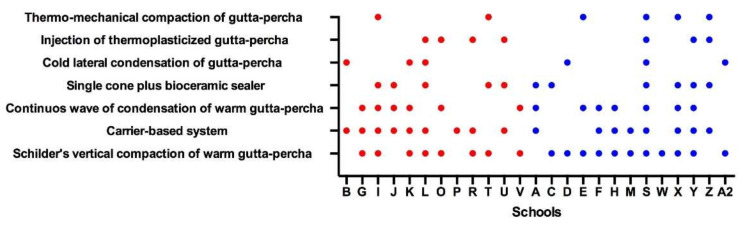
Obturation techniques used during clinical training. Schools belonging to Groups 1 and 2 are represented by red and blue dots, respectively.

The frequency of the obturation techniques used during clinical training is presented in Figure 4.

A total of 14 out of 25 schools responded that, whenever possible, root canal treatment was carried out in a single visit. When an interappointment dressing was inserted into the canals, all schools used calcium hydroxide. One school also reported the use of Cresatina (OGNA S.r.l, Muggiò, Italy).

A total of 18 out of 24 schools (75.0%) (8/12 in Group 1, and 10/12 in Group 2, not significant) did not require a minimum number of canals to be treated by students during clinical training. In the remaining schools, the minimum number of canals to be treated ranged from 5 to 30, with a mean of 12.7 ± 9.5 (14.5 ± 9.7 in Group 1, and 9 ± 7.3 in Group 2, not significant).

## 4. Discussion

This was the first survey to investigate endodontic teaching in Italian dental schools. Although 8 out of 36 schools did not participate, the data gathered are representative and can be used to outline and compare the current standard of endodontic teaching within Italian dental schools. Diversity among schools is, to a degree, unavoidable due to the specificities of each school (location, staff involved, number of students, and economic resources), but a harmonisation of the curricula is highly desirable. Despite variability in the responses, some general aspects shared by almost all of the schools could be identified. The endodontic curriculum was composed of three main activities: lectures and the associated learning activities, preclinical training, and clinical training. Lectures and the associated learning activities should provide robust knowledge for the safe practice of endodontics. The most important endodontic topics were addressed, but with this kind of research, it was not possible to ascertain the efficacy of the theoretical teaching.

Preclinical training is a fundamental learning activity in which students should become familiar with the procedures and techniques required to transit comfortably into clinical settings. The ESE undergraduate curriculum guidelines for endodontology [11] do not set a minimum number of hours of preclinical training, and among the surveyed schools, the number of hours of preclinical training ranged from 4 to 100. While a total of 4 h is probably insufficient to reach the learning objectives, most schools spent 20–40 h in preclinical training, which seems to be adequate. In preclinical training, the recently introduced 3D-printed artificial dental simulators overcome some limitations of extracted teeth (e.g., availability, cross-infection control, ethical issues, uniformity), but natural teeth are still superior in replicating clinical reality and, accordingly, were used in most preclinical endodontic training courses.

In only three schools was clinical training not provided. We do not know whether that was due to the incorrect interpretation of the question or due to a temporary restriction during the pandemic; in either case, it would be strongly recommended that efforts be made to implement clinical training in these schools. According to the ESE guidelines [11], the student should be competent at treating uncomplicated anterior and posterior teeth. This means that clinical training in which students exclusively observe during clinical sessions, without directly delivering the endodontic treatments, as reported by two schools, hardly makes the graduating dentist capable of carrying out treatments without supervision.

Endodontic courses in which the leader was an academic-structured figure (full or associate professor) showed some statistically significant differences in the considered variables as compared to courses led by researchers or temporarily lecturers. In Group 1 schools, endodontics was more often taught over several academic years, more hours were allocated to teaching the topic “Nonsurgical root canal treatment”, a greater number of collaborators were involved in teaching activities, and a greater number of students attended the courses. Group 1 schools devoted almost twice as much time to preclinical training and more frequently had a clinical area specifically assigned to endodontics as compared to Group 2; however, these differences were not statistically significant. These data cannot lead us to state that students attending courses in Group 1 schools are better trained than students attending courses in Group 2 schools because such a conclusion would require a more in-depth evaluation. It is reasonable to think that teachers who reach the highest levels in their academic careers are generally those with more experience and a demonstrated ability to achieve goals in their work. High-level professors acquire an important weight on the institutional boards that define the study plans, and they are able to raise funds for their research and educational activities as well. Full and associate professors coordinate teams of people working together for research, clinical assistance, and education. Therefore, it is not surprising that the endodontic courses in the schools of Group 1 share some positive aspects with those of Group 2.

The possibility of European dentists moving freely within European countries demands the convergence of the undergraduate curriculum and quality teaching assurance [16]. Several studies evaluated undergraduate endodontic programs in different European countries [12,13,16,17,18,19]. A direct comparison of these studies is prevented by two main limitations: the surveys were carried out at different times (up to 19 years apart), and the questionnaires were different. We used a questionnaire that is almost identical to those used recently in the UK and Spain [12,13]; thus, we can reliably compare the educational systems in these three countries. Italy has more dental schools per million inhabitants than either Spain or the United Kingdom. On the one hand, this allows for smaller classes and students who can be better tutored; on the other hand, such a setting leads to a dilution of resources.

In Italy, students attend the endodontic course later in the degree program as compared to those in both the United Kingdom and Spain, where endodontics is mainly taught in the 3rd and 4th years, respectively. Scheduling the endodontics course 1 year earlier would lend the advantage of preserving more time for the clinical internship, which is usually carried out in the last years of the degree program. The teaching methods are similar to those used in England and Spain, with the exception of problem-based learning, which is seldom used in Italy.

In Italy, the mean number of hours dedicated to teaching the major subject, namely “Nonsurgical endodontic treatment”, was lower compared that in both the United Kingdom and Spain. Conversely, the mean number of hours dedicated to preclinical training was greater compared to that in Spain. According to our results, the average number of hours devoted to clinical training was 84.1, and 88% and 56% of the schools devoted more than 20 and 50 h, respectively. These data are similar to those recorded in Spain, where 95% of schools devoted more than 20 h, and 60% more than 50 h; the percentage of schools that devoted more than 20 h to clinical training was lower in the UK (73%).

As already stated, since the ESE guidelines do not define a minimum number of hours for teaching every single subject and for preclinical training, each school is left free to decide the number of hours to allocate to different learning activities.

The average student:staff ratio was similar to that in the UK and Spain. In Italy, Schilder’s technique [20] is widely taught, contrary to cold lateral compaction, which is indeed more popular in English and Spanish schools. Since no obturation technique has been demonstrated to be superior [21], the choice of technique is directly influenced by the preference of the teacher. Italian schools of endodontics are traditionally oriented to teach Schilder’s technique [22].

## 5. Conclusions

The data provided in this study are the first to be collected on the teaching of endodontics in Italy. This kind of study is useful for promoting the harmonisation of curricula. Educators should strive to make the teaching programs more uniform by following the ESE Guidelines and to improve clinical training.

## Figures and Tables

**Table 1:** Number of students attending the course.

Number of Students	Number of Schools (%)
1–14	4 (14.3)
15–29	14 (50)
30–44	7 (25)
45–49	2 (7.1)
>60	1 (3.6)

## Data Availability

The data presented in this study are available upon request from the corresponding author.

## Supplementary Materials

The following supporting information can be downloaded at: www.mdpi.com/article/10.3390/jcm11237190/s1, File S1. Questionnaire adapted from Al Raisi et al., 2019 [12]; File S2. Answers to the questions “In which year/years are the specific topics of the endodontics program taught?”.
