# Supplementary material for: How Is Endodontics Taught in Italy? A Survey of Italian Dental Schools
Source: J Clin Med. 2022 Dec 2;11(23):7190. doi: 10.3390/jcm11237190 (PMC9738303; doi:10.3390/jcm11237190)

Supporting information

Supplemental material File S1

Questionnaire adapted from Al Raisi et al., 2019 [12].

1. What learning activities does the Endodontics course consist of? (it is possible to mark more than one answer)
   - ☐ Lectures
   - ☐ Pre-clinical training
   - ☐ Clinical training
   - ☐ Others ____________________________

2. In what year/years is endodontics taught? (it is possible to mark more than one answer)
   - ☐ First year
   - ☐ Second year
   - ☐ Third year
   - ☐ Fourth year
   - ☐ Fifth year
   - ☐ Sixth year

3. What teaching methods are used? (it is possible to mark more than one answer)
   - ☐ Frontal lectures
   - ☐ Seminars
   - ☐ Study group
   - ☐ Indipendent study
   - ☐ Projects
   - ☐ E-learning
   - ☐ Video
   - ☐ Manuals
   - ☐ Reading lists
   - ☐ Problem based learning
   - ☐ Other ____________________________

4. In which year/years are the specific topics of the Endodontics program taught? (it is possible to mark more than one answer)

	First	Second	Third	Fourth	Fifth	Sixth	Not taught
Root canal anatomy and pulp histology	☐	☐	☐	☐	☐	☐	☐
Pulp physio-pathology	☐	☐	☐	☐	☐	☐	☐
Endodontic microbiology	☐	☐	☐	☐	☐	☐	☐

Endodontic radiology	☐	☐	☐	☐	☐	☐	☐
Endodontic materials	☐	☐	☐	☐	☐	☐	☐
Vital pulp therapy	☐	☐	☐	☐	☐	☐	☐
Non surgical endodontic treatment	☐	☐	☐	☐	☐	☐	☐
Root canal treatment of immature teeth	☐	☐	☐	☐	☐	☐	☐
Retreatments	☐	☐	☐	☐	☐	☐	☐
Surgical endodontics	☐	☐	☐	☐	☐	☐	☐
Regenerative endodontics	☐	☐	☐	☐	☐	☐	☐
Post-endodontic restoration	☐	☐	☐	☐	☐	☐	☐
Bleaching of endodontically treated teeth	☐	☐	☐	☐	☐	☐	☐
Dental trauma and emergencies	☐	☐	☐	☐	☐	☐	☐

5. What is the number of hours allocated for teaching the following topics?
   - Root canal anatomy and pulp histology
   - Pulp physio-pathology and endodontic microbiology
   - Endodontic radiology
   - Endodontic materials
   - Vital pulp therapy
   - Non surgical endodontic treatment
   - Root canal treatment of immature teeth
   - Retreatments
   - Surgical endodontics
   - Regenerative endodontics
   - Post-endodontic restoration
   - Bleaching of endodontically treated teeth

- Dental trauma and emergencies

6. What is the qualification of the course leader?
   - ☐ Full professor
   - ☐ Associate professor
   - ☐ Researcher
   - ☐ Temporarily lecturer
   - ☐ Other ___________________________

7. Which is the main clinical activity of the course leader?
   - ☐ General dentistry
   - ☐ General dentistry with particular interest in endodontics
   - ☐ Exclusively Endodontics
   - ☐ Other___________________________

8. Are there any collaborators in addition to the course manager? If so, how many? What qualification do they have?

---

9. How many students attend the course?
   - ☐ 1-14
   - ☐ 15-29
   - ☐ 30-44
   - ☐ 45-59
   - ☐ 60-74
   - ☐ 75-89
   - ☐ 90-104
   - ☐ Più di 104

10. What is the staff:students ratio during pre-clinical training?
    ____

11. What is the staff:students ratio during clinical training?
    ____

12. What types of endodontic treatments do students perform during pre-clinical training? (it is possible to mark more than one answer)
    - ☐ Root canal treatment of single-rooted teeth
    - ☐ Root canal treatment of multi-rooted teeth
    - ☐ Vital pulp therapy
    - ☐ Endodontic surgery
    - ☐ Regenerative endodontics
    - ☐ Bleaching of endodontically treated teeth
    - ☐ Retreatments

13. What types of endodontic treatments do students perform during clinical training? (it is possible to mark more than one answer)
    - ☐ Root canal treatment of single-rooted teeth

- ☐ Root canal treatment of multi-rooted teeth
- ☐ Vital pulp therapy
- ☐ Endodontic surgery
- ☐ Regenerative endodontics
- ☐ Bleaching of endodontically treated teeth
- ☐ Retreatments
- ☐ Students do not execute any treatment

14. How many hours are allocated to pre-clinical training?

    ____

15. What is the average cost in euro that each student has to endure to purchase the material to be used in the pre-clinical training?

    ____

16. How many hours are allocated to clinical training?

    ____

17. Which teeth are treated by the students during the pre-clinical training? (it is possible to mark more than one answer)
    - ☐ Incisors
    - ☐ Canines
    - ☐ Premolars
    - ☐ Molars

18. Which teeth are treated by the students during the pre-clinical training? (it is possible to mark more than one answer)
    - ☐ Incisors
    - ☐ Canines
    - ☐ Premolars
    - ☐ Molars

19. What types of root canals are used in pre-clinical training? (it is possible to mark more than one answer)
    - ☐ Canals in natural extracted teeth
    - ☐ Canals in commercial plastic teeth
    - ☐ Canals in 3D printed teeth
    - ☐ Canals in acrylic blocks with simple curves
    - ☐ Canals in acrylic blocks with S-shaped curves
    - ☐ Other _________________

20. It is present a clinical area specifically assigned to Endodontics?
    - ☐ Yes
    - ☐ No

21. Which magnifying systems are used by students during pre-clinical training? (it is possible to mark more than one answer)
    - ☐ Magnifying systems are not used

- ☐ Galilean/prismatic loops
- ☐ Microscope
- ☐ Other __________________

22. Which magnifying systems are used by students during clinical training? (it is possible to mark more than one answer)
    - ☐ Magnifying systems are not used
    - ☐ Galilean/prismatic loops
    - ☐ Microscope
    - ☐ Other __________________

23. Do students use ultrasonic instruments?
    - ☐ Yes, only during pre-clinical training
    - ☐ Yes, only during clinical training
    - ☐ Yes, both during pre-clinical and clinical training
    - ☐ No

24. What method of working length determination is used during clinical training?
    - ☐ Radiographs
    - ☐ Electronic apex locator
    - ☐ Radiographs + electronic apex locator
    - ☐ Other __________________

25. Which instruments are used for root canal shaping during pre-clinical training? (it is possible to mark more than one answer)
    - ☐ Manual stainless instruments
    - ☐ Engine driven rotary NiTi files
    - ☐ Engine driven reciprocanting NiTi files

26. Which instruments are used for root canal shaping during pre-clinical training? (it is possible to mark more than one answer)
    - ☐ Manual stainless instruments
    - ☐ Engine driven rotary NiTi files
    - ☐ Engine driven reciprocanting NiTi files

27. Which irrigants are used during clinical training? (it is possible to mark more than one answer)
    - ☐ None
    - ☐ Water
    - ☐ Saline
    - ☐ Local anaesthestic solution
    - ☐ Sodium hypochlorite
    - ☐ Chlorhexidine
    - ☐ EDTA
    - ☐ Other __________________

28. Which method of root canal filling is used during pre-clinical training? (it is possible to mark more than one answer)

- ☐ Single cone technique
- ☐ Cold lateral condensation
- ☐ Vertical hot condensation
- ☐ Thermoplastic injection technique
- ☐ Carrier-based technique
- ☐ Paste fillers
- ☐ Continuous wave of condensation
- ☐ Thermo-mechanic condensation
- ☐ Other _________________

29. Which method of root canal filling is used during clinical training? (it is possible to mark more than one answer)
    - ☐ Single cone technique
    - ☐ Cold lateral condensation
    - ☐ Vertical hot condensation
    - ☐ Thermoplastic injection technique
    - ☐ Carrier-based technique
    - ☐ Paste fillers
    - ☐ Continuous wave of condensation
    - ☐ Thermo-mechanic condensation
    - ☐ Other _________________

30. Which type of endodontic dressings are used during clinical training? (it is possible to mark more than one answer)
    - ☐ Calcium hydroxide
    - ☐ None – canals are left empty in between appointments
    - ☐ None – single visit treatment whenever possible
    - ☐ Other _________________

31. Is there a minimum number of canals that students have to treat during pre-clinical training?
    - ☐ Yes (how many?)
    - ☐ No

32. Is there a minimum number of canals that students have to treat during clinical training?
    - ☐ Yes (how many?)
    - ☐ No

Supplemental material File S2

Answers to the questions “In which year/years are the specific topics of the Endodontics program taught? (it is possible to mark more than one answer)”

"Root canal anatomy and pulp histology"

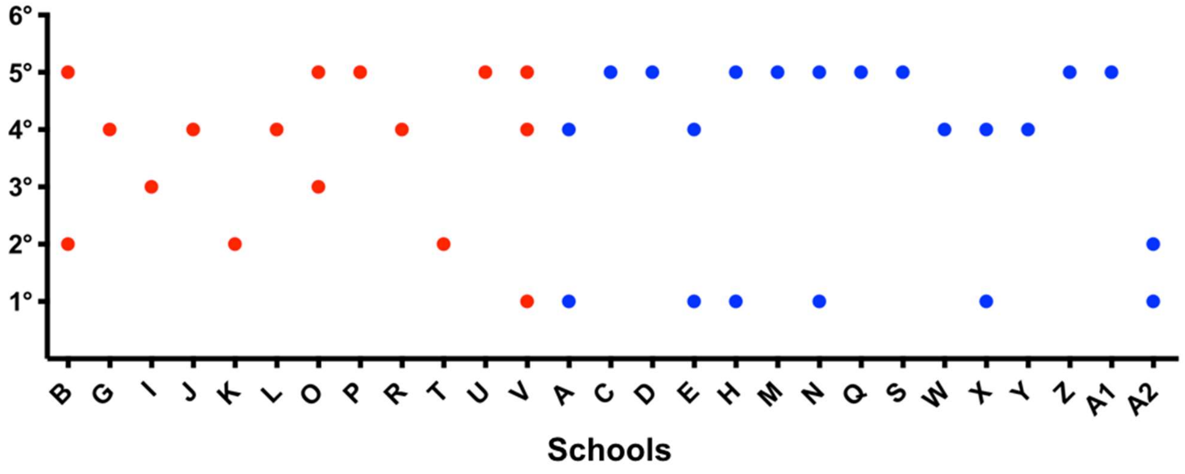

"Pulp physio-pathology"

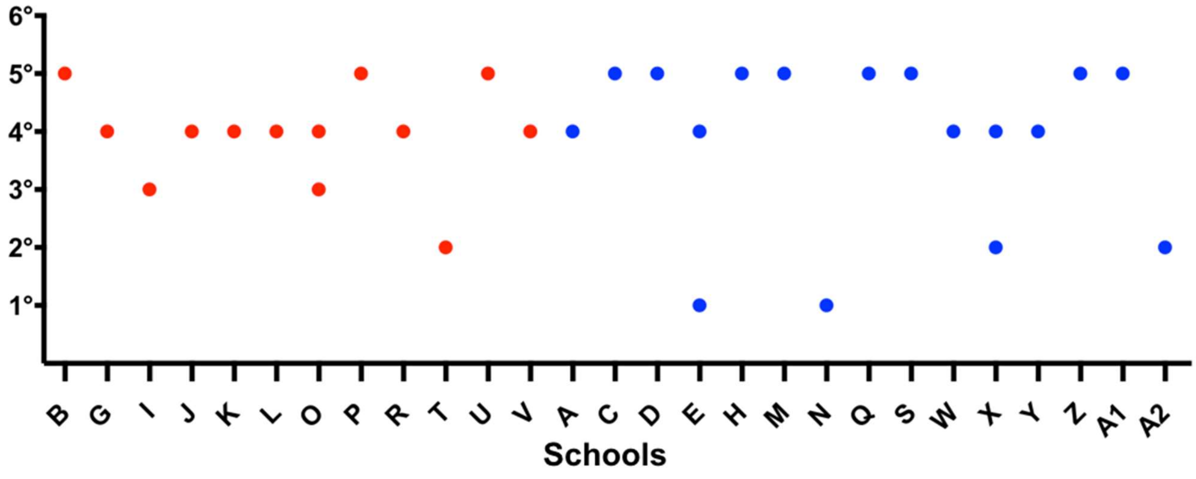

"Endodontic microbiology"

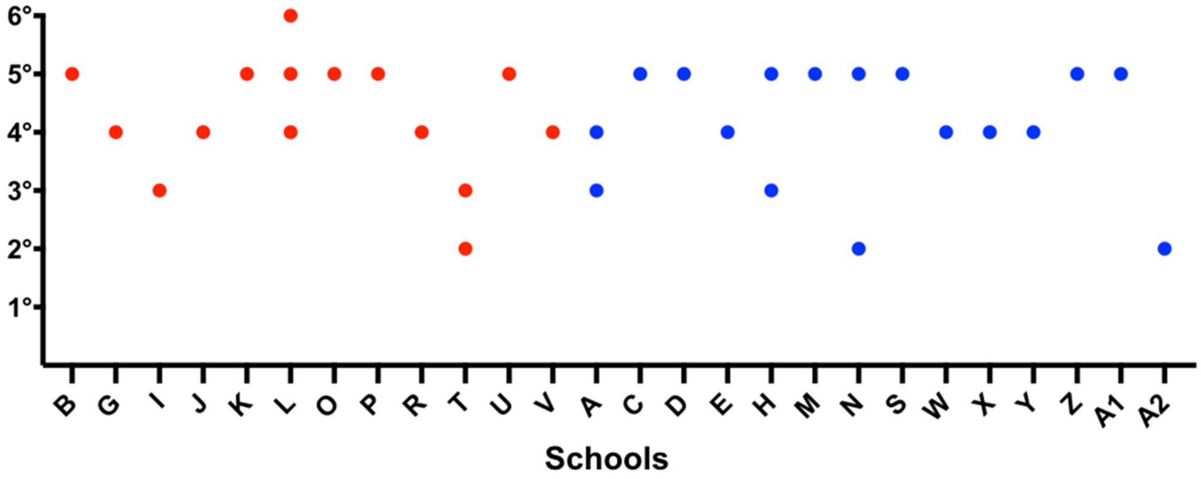

"Endodontic radiology"

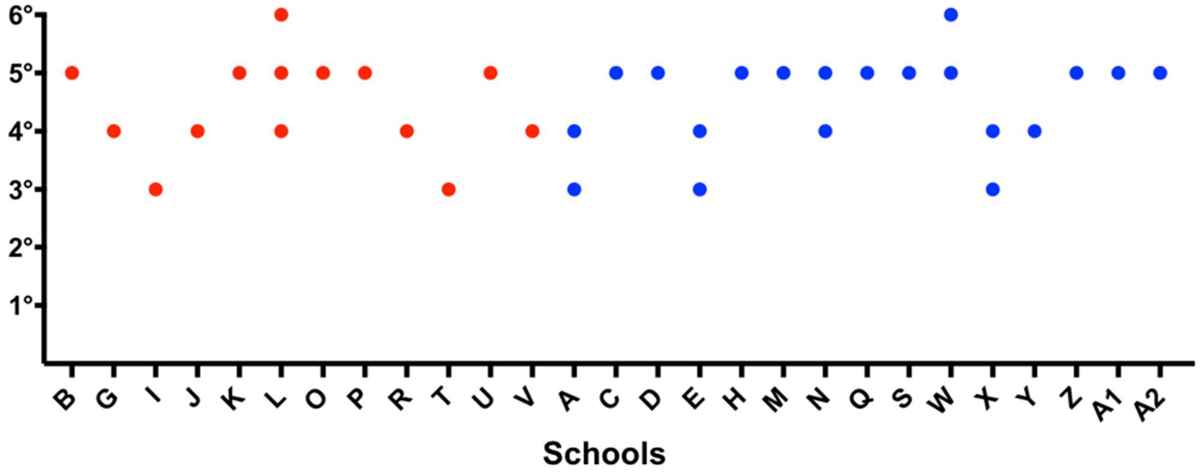

"Endodontic materials"

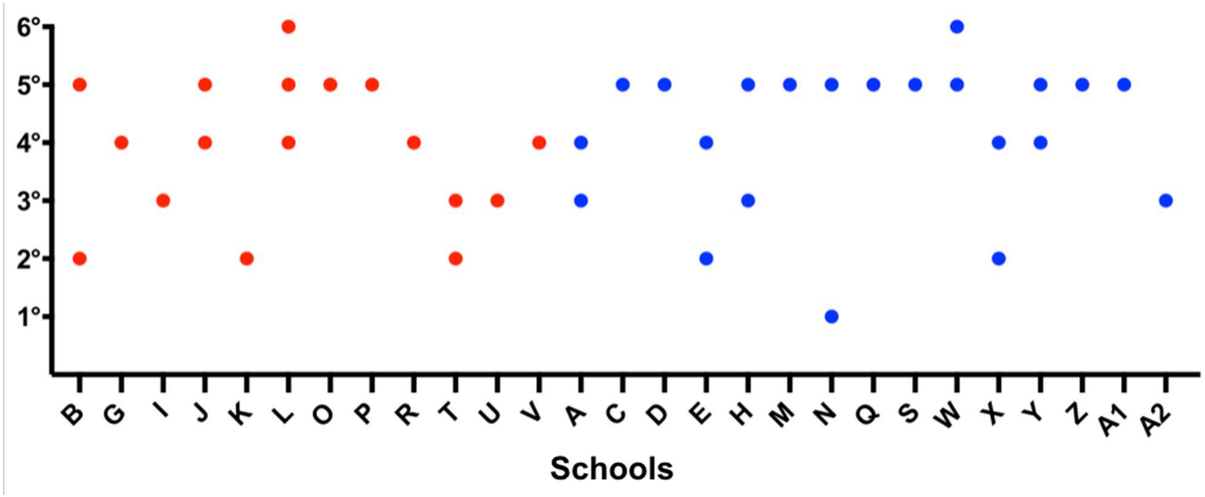

"Vital pulp therapy"

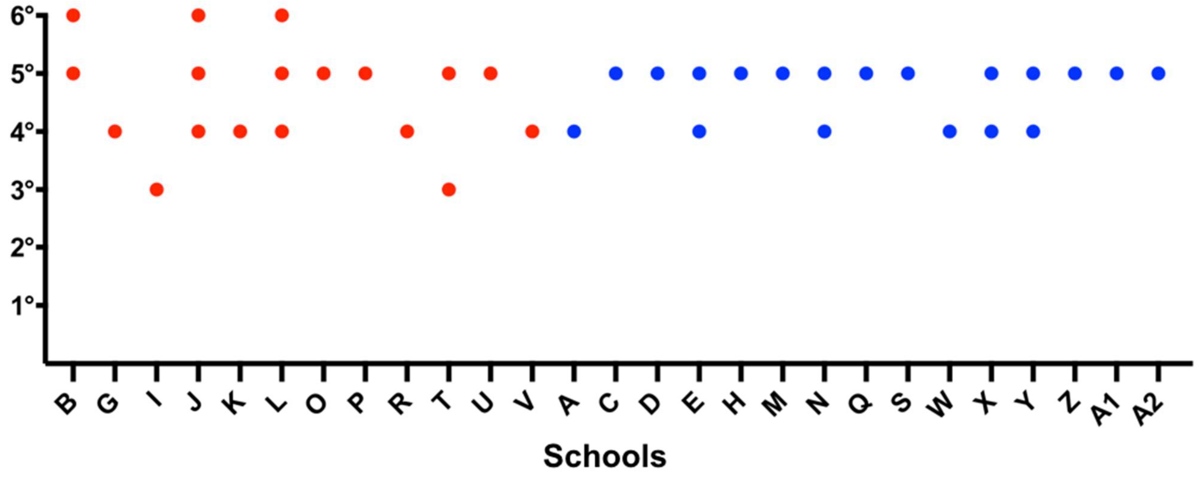

"Root canal treatment"

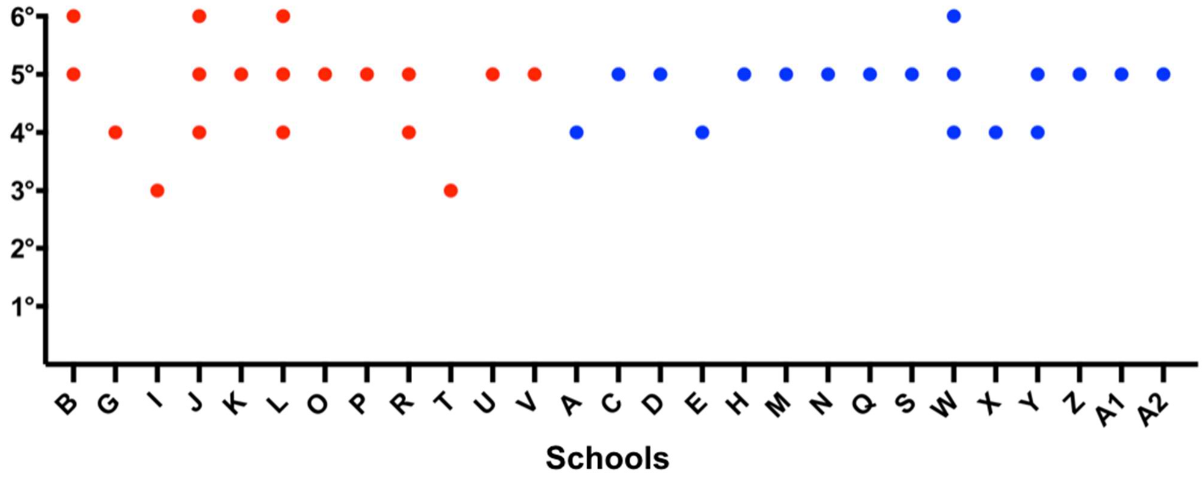

"Root canal treatment of immature teeth"

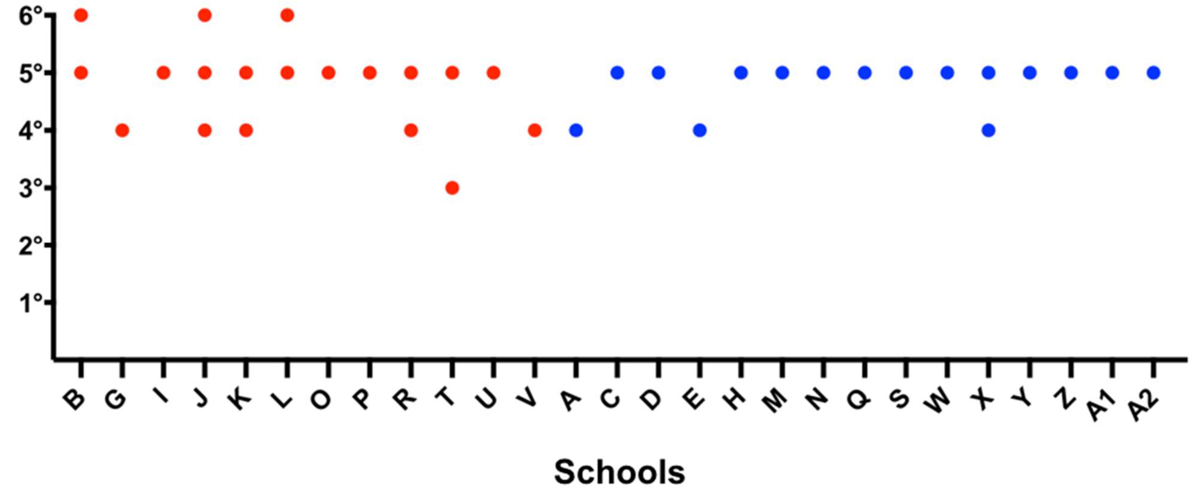

"Retreatment"

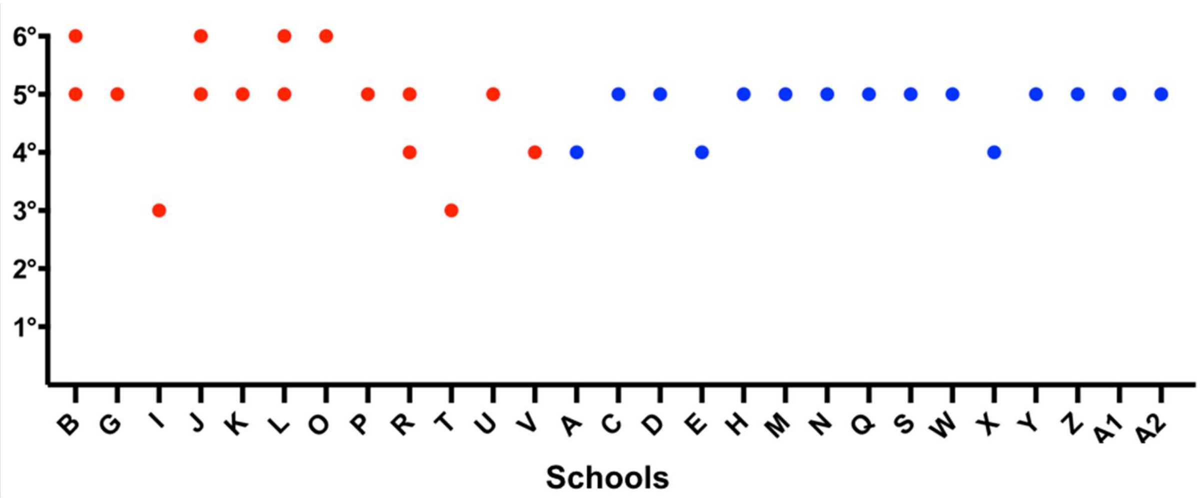

"Endodontic surgery"

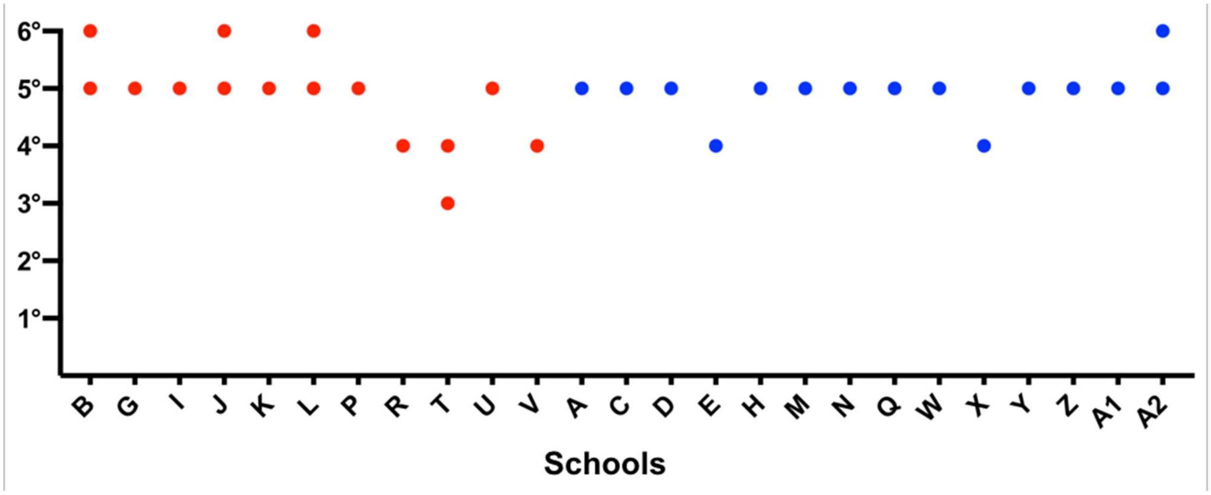

"Regenerative endodontics"

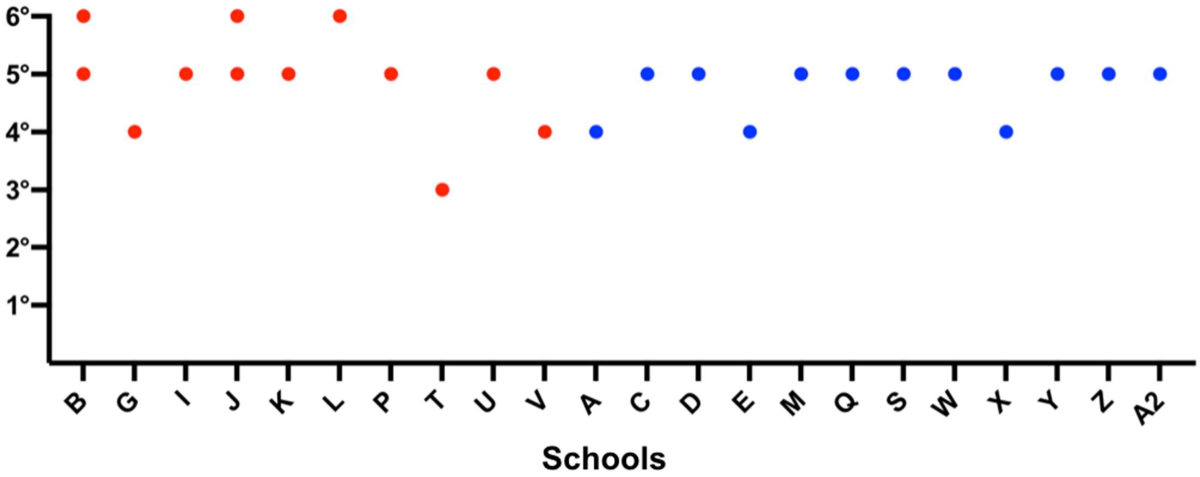

"Post-endodontic restoration"

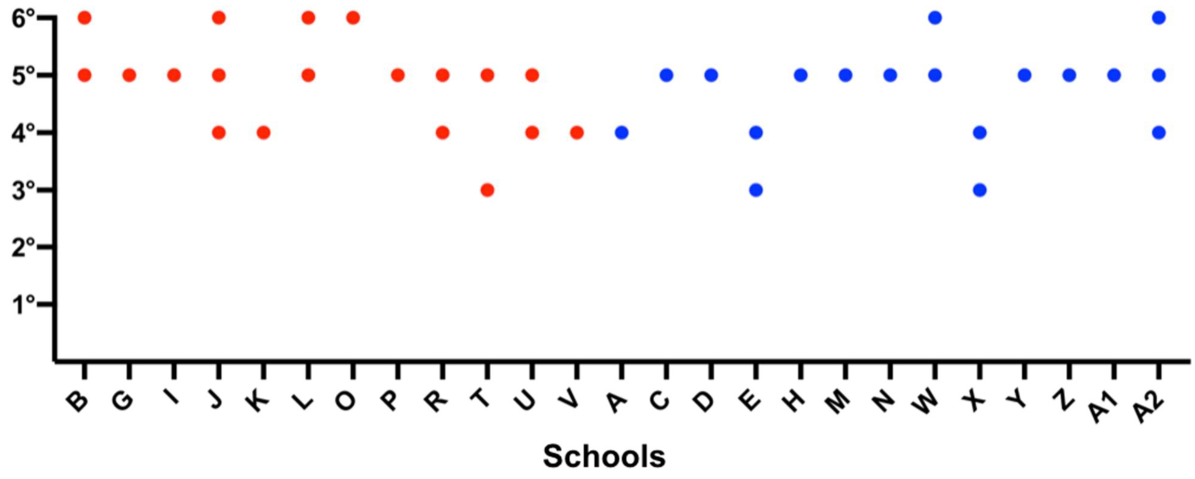

"Bleaching of endodontically treated teeth"

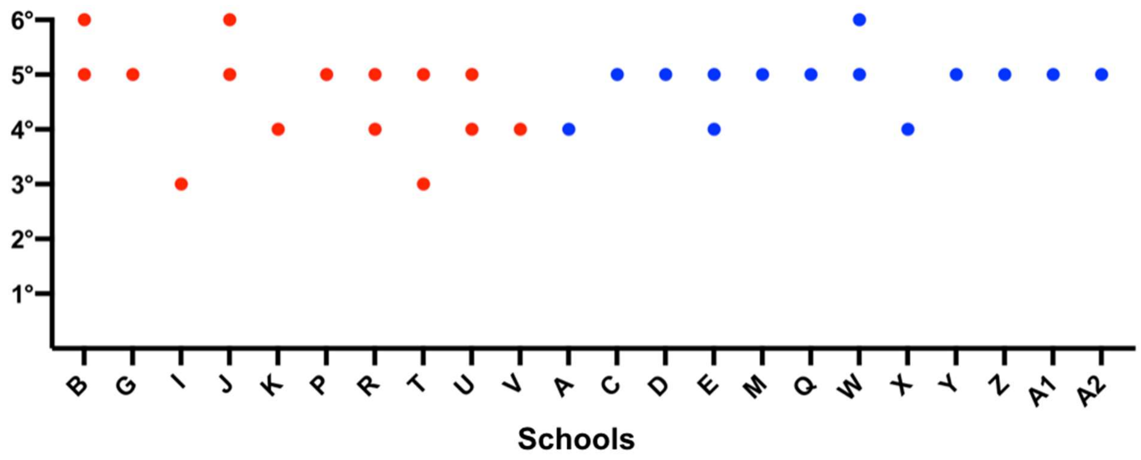

"Dental trauma and emergencies"

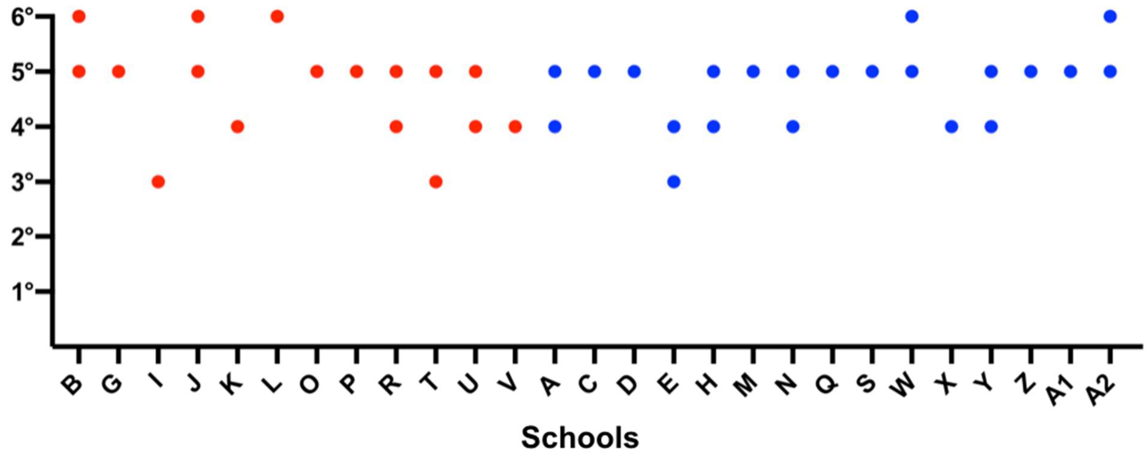

Supplement: Supplementary file 1 [file jcm-11-07190-s001.zip › jcm-2057860-supplementary.pdf]
